# A novel homozygous variant (c.5876T > C: p. Leu1959Pro) in *DYSF* segregates with limb-girdle muscular dystrophy: a case report

**DOI:** 10.1186/s12891-024-07354-9

**Published:** 2024-03-27

**Authors:** Hamed Hesami, Serwa Ghasemi, Golnaz Houshmand, Yalda Nilipour, Mahshid Hesami, Alireza Biglari, Shahriar Nafissi, Majid Maleki, Samira Kalayinia

**Affiliations:** 1 Rajaie Cardiovascular Medical and Research Center, Iran University of Medical Sciences, Tehran, Iran; 2 Cardiogenetic Research Center, Rajaie Cardiovascular Medical and Research Center, Iran University of Medical Sciences, Tehran, Iran; 3 Pediatric Pathology Research Center, Research Institute for Children’s Health, Shahid Beheshti University of Medical Sciences, Tehran, Iran; 4 Neuromuscular Research Center, Tehran University of Medical Sciences, Tehran, Iran; 5 School of Medicine, Tehran University of Medical Sciences, Tehran, Iran

**Keywords:** *DYSF*, Limb-girdle muscular dystrophy, Dysferlin, Whole-exome sequencing

## Abstract

**Background:**

Limb girdle muscular dystrophies (LGMDs) constitute a heterogeneous group of neuromuscular disorders with a very variable clinical presentation and overlapping traits. The clinical symptoms of LGMD typically appear in adolescence or early adulthood. Genetic variation in the dysferlin gene (*DYSF*) has been associated with LGMD.

**Methods:**

We characterized a recessive LGMD in a young adult from consanguineous Irani families using whole-exome sequencing (WES) technology. Sanger sequencing was performed to verify the identified variant. Computational modeling and protein-protein docking were used to investigate the impact of the variant on the structure and function of the DYSF protein.

**Results:**

By WES, we identified a novel homozygous missense variant in *DYSF* (NM_003494.4: c.5876T > C: p. Leu1959Pro) previously been associated with LGMD phenotypes.

**Conclusions:**

The identification and validation of new pathogenic DYSF variant in the present study further highlight the importance of this gene in LGMD.

**Supplementary Information:**

The online version contains supplementary material available at 10.1186/s12891-024-07354-9.

## Introduction

A range of muscle disorders known as dysferlinopathy are distinguished by two major phenotypes, namely Miyoshi muscular dystrophy and LGMD type R2, in addition to two lesser phenotypes, Distal myopathy and asymptomatic hyperCKemia with anterior tibial onset [1].

- Muscle atrophy and weakening are the usual signs of Miyoshi muscular dystrophy, which usually appears around the age of 19. The condition mostly affects the distal regions of the legs, especially the gastrocnemius and soleus muscles. The thigh and gluteal muscles eventually become involved in this weakening as it advances.
- During adolescence or early adulthood, LGMD type R2 first manifests as early weakening and atrophy of the pelvic and shoulder-girdle muscles, with a sluggish progression.
- Asymptomatic hyperCKemia is characterized solely by a marked elevation in serum creatine kinase (CK) concentration.
- The hallmark of distal myopathy with anterior tibial start is an early and predominate weakening in the distal muscles, especially those in the legs’ anterior compartment.

Limb-girdle muscular dystrophy (LGMD) encompasses a broad range of genetic disorders characterized by progressive muscle weakness and dystrophic changes in muscle tissue. Genetic variations that cause these illnesses can be inherited in either an autosomal dominant or recessive fashion [2]. LGMD symptoms can arise at any age, however they usually do so in adolescence or early adulthood. Common symptoms include difficulty rising from a seated or prone position, climbing stairs, and lifting objects due to weakness in the upper arms and thighs. LGMD can also affect other body systems, including cardiovascular and respiratory systems [3]. Variants in the dysferlin gene (*DYSF*), which encode DYSF (MIM*603,009), are associated with an autosomal recessive disorder characterized by various clinically distinct muscular dystrophies, including LGMD [4]. *DYSF*, is located on chromosome 2p13 and consisting of 55 exons, encodes a calcium-dependent transmembrane protein abundantly expressed in skeletal and heart muscles. Dysferlin is involved in mechanisms related to cell membrane repair [5]. Currently, next-generation sequencing (NGS) is considered the gold standard for precise diagnosis of various myopathies [6]. The MYO-SEQ project conducted whole-exome sequencing (WES) on 1001 patients diagnosed with limb-girdle muscle disease or unexplained limb-girdle weakness, identifying pathogenic variants in *DYSF*, causing LGMD type R2 or dysferlinopathy, in 45 cases (4.5%) [7]. A Polish study performed WES on 73 patients clinically diagnosed with LGMD, identifying pathogenic *DYSF* variants causing LGMD type 2B or a dysferlinopathy subtype of LGMD in 6 cases (8%). These findings demonstrated that mutations in *DYSF* underlie the pathogenesis of the disease in a subset of LGMD patients [8].

In this study, we employed WES to accurately diagnosis a patient with muscular dystrophy, previously misdiagnosed based on routine clinicopathological examinations. Here, we report a novel homozygous variant in *DYSF* likely associated with the myopathy phenotype within the patient’s family.

## Materials and methods

### Patient’s clinical history

The proband was the eldest child born into a healthy family of five individuals. Although the parents were consanguineous (cousins), his younger brother and sister showed no abnormalities or signs of the same illness. The patient remained in good health until the age of 22 when he began experiencing difficulty walking, weakness in the legs, and dystrophy in the lower limbs. The weakness initially appeared bilaterally in the toes and progressed to involve the calf muscles and distal regions of the lower limbs, while upper limb strength remained unaffected. Upon visiting a local hospital, the patient underwent a thorough examination and was diagnosed with weakness, subsequently receiving supportive treatment. Further diagnostic evaluations at better-equipped facilities were recommended for a comprehensive understanding of the condition. He was managed conservatively with a treatment plan aimed at alleviating symptom, with referral to a specialized tertiary care center advised for additional advanced testing and access to resources necessary for establishing a definitive diagnosis. Supportive therapy remained the cornerstone of the treatment plan in the interim.

The patient’s serum CK levels were elevated at 2.901 U/L. Neurological examinations and evaluations, including electromyography, indicated an inflammatory myopathic process. Biopsy of the right vastus lateralis muscle revealed an atrophic pattern, prompting a recommendation for genetic studies. At the age of 24, the patient contracted COVID-19 and subsequently recovered; nonetheless, he continued to experience dyspnea, leading to a referral to Rajaie Cardiovascular Medical and Research Center, Tehran, Iran, for further evaluation. At the hospital, an echocardiographic examination revealed a decreased left ventricular ejection fraction (20%), prompting the medical team advised a cardiac magnetic resonance imaging scan.

The present study adheresto the Declaration of Helsinki, and ethical approval was obtained from the Ethics Committees of Rajaie Cardiovascular Medical and Research Center, Iran University of Medical Sciences, Tehran, Iran (IR.RHC.REC.1402.033). Written informed consent was obtained from the study participants.

#### Muscle biopsy

**Fig. 1:**
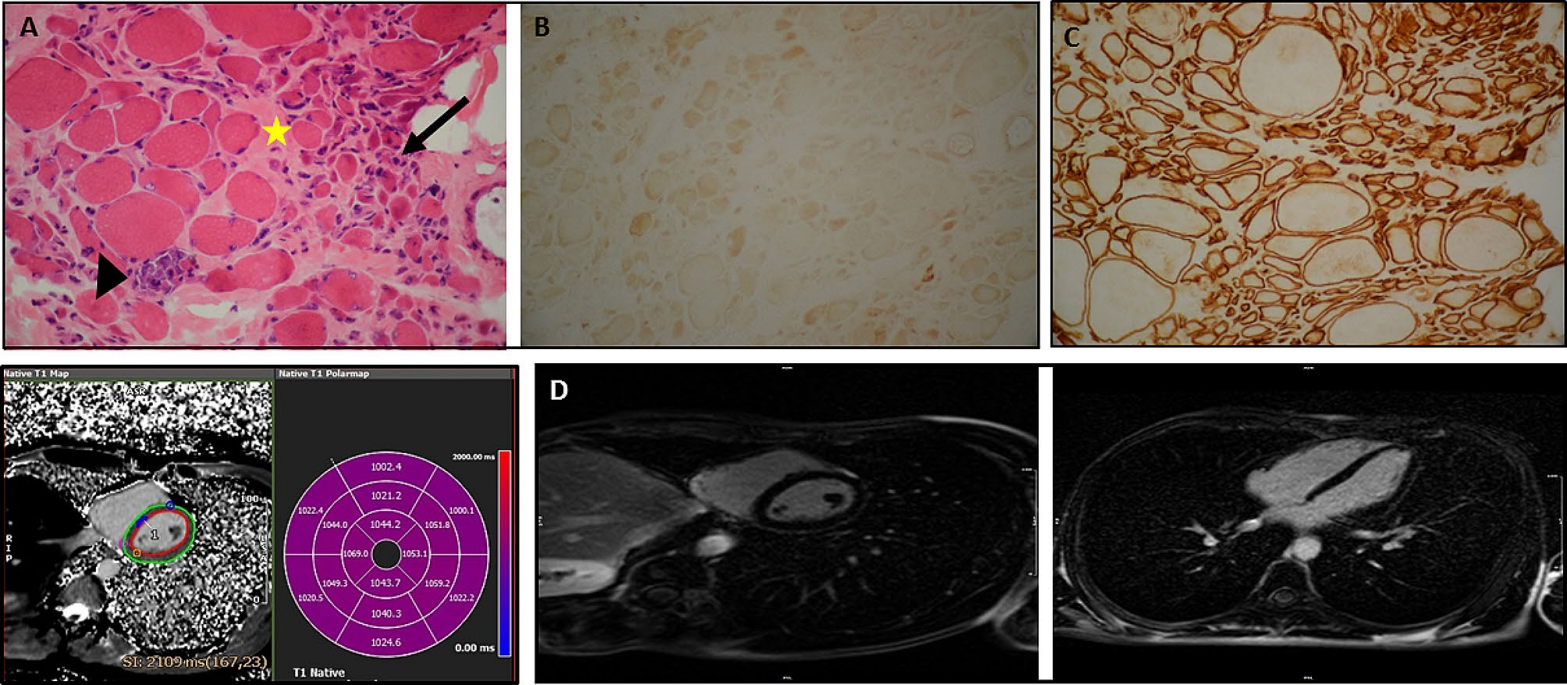
(**A**) The image shows the prominent group atrophy pattern of round and angular muscle fibers with a single focus on myophagocytosis and severe endomysial fibrosis (x400). (**B**) The image demonstrates the loss of sarcolemmal labeling of all muscle fibers with the dysferlin (DYSF) antibody (x400) with a positive control with the β-spectrin antibody (x400). (**D**) The image presents the T1 mapping image and the bullseye plot, showing T1 map segmental values (mid-septal T1 value = 1040 ms). (**D**) The image illustrates the late enhancement images at the mid-level short-axis view and the 4-chamber view, respectively, showing no replacement fibrosis. (**E**) The image shows that the Leu1959 residue is highly conserved throughout species according to the multiple sequence alignment of DYSF sequences

A muscle biopsy via the open technique from the patient’s right vastus lateralis was requested (Fig. 1A). Histochemical and immunohistochemical studies were performed on the fresh muscle sample, frozen in isopentane cooled in liquid nitrogen (Fig. 1B-C). The panel of stains was composed of hematoxylin and eosin, modified Gomori Trichrome, PAS, Oil red O, Congo Red, NADH-TR, SDH, Cox, Cox + SDH, and ATPase. Additionally, an immunohistochemical study was performed with dystrophins, sarcoglycans, DYSF, merosin, and β-spectrin antibodies from Novocastra.

#### Cardiac magnetic resonance imaging

First, a cardiac magnetic resonance imaging test (1.5T MAGNETOM Sola, Siemens Healthcare, Erlangen, Germany) was performed to assess left ventricular myocardial function. Next, a mass steady-state free precession functional imaging test with breath-holding was performed in the long-axis (4-, 3-, and 2-chamber) views and 10 to 12 consecutive short-axis stacks. Right ventricular outflow and inflow-outflow views were also acquired. Afterward, short Tau inversion recovery sequences with breath-holding were taken in the long- (2-, 3-, and 4-chamber) and short-axis views to assess edema and inflammation. Subsequently, T1 mapping was acquired using a 17-heartbeat steady-state free precession, single-breath-hold modified Look-Locker inversion recovery sequence in 3 short-axis (base, mid-cavity, and apex) sequences before contrast administration to evaluate interstitial fibrosis by T1 values of the myocardium. Then, contrast administration was performed with 0.15 mmol/kg of gadoterate meglumine (gadolinium-DOTA, Dotarem, Guerbet SA, Paris, France) to assess myocardial fibrosis. Finally, the magnitude and phase-sensitive inversion recovery reconstructions of early and late gadolinium images were taken in the short-axis stack (the 4-, 3-, and 2-chamber views) (Fig. 1D).

### Genetic analysis

#### DNA isolation and WES

**Fig. 2:**
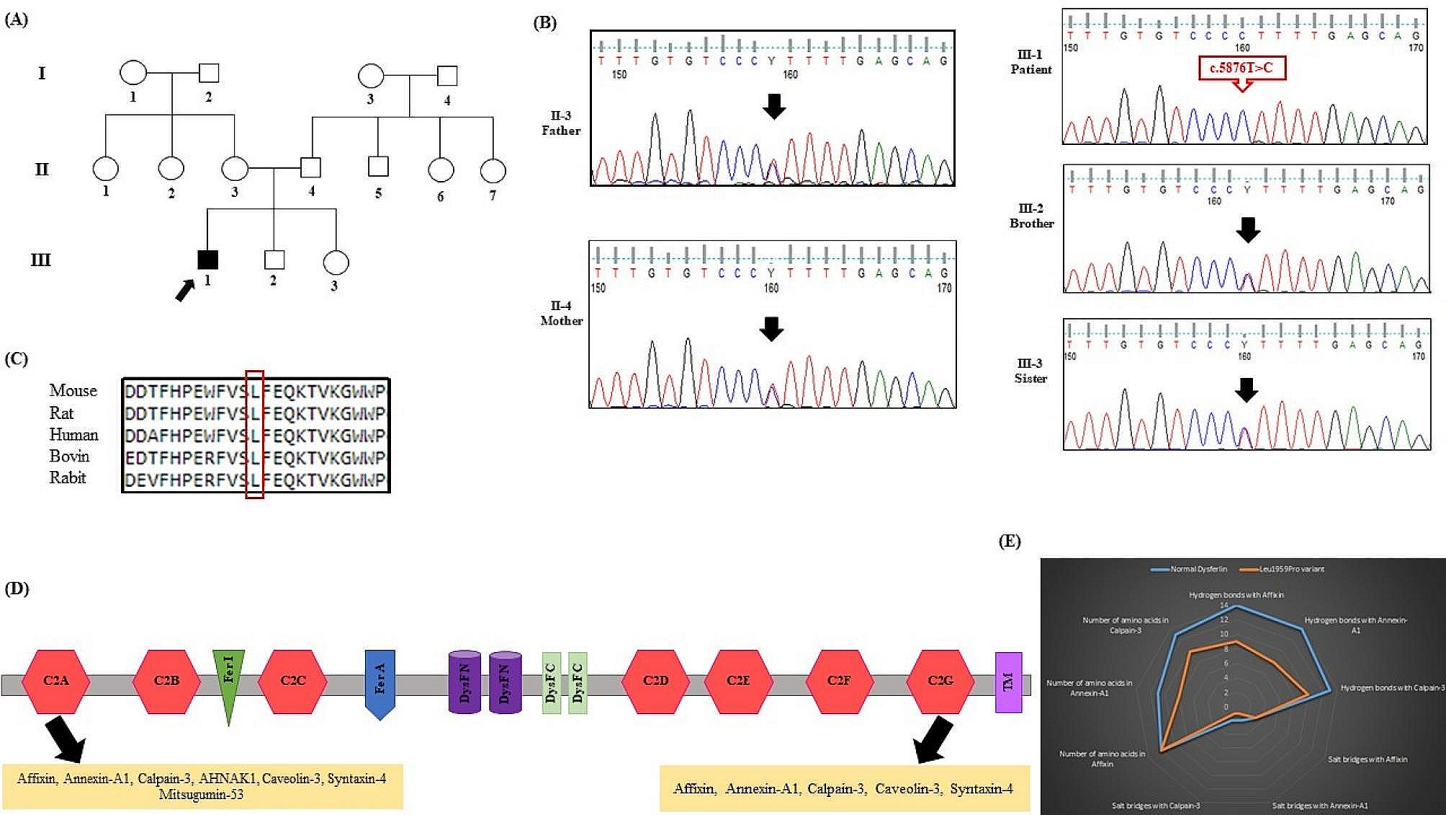
The pathogenic variant of *DYSF* is linked to limb-girdle muscular dystrophy (LGMD). (**A**) A family pedigree displaying the DYSF-affected family is presented. Variant carriers are represented in black, while relatives without the variant are in white. The squares denote males, the circles indicate females, and the arrow points to the proband. (**B**) Sanger-sequencing chromatograms reveal the *DYSF* variant sequence in the father, mother, sister, brother, and their DYSF-affected son. The arrow highlights the T/C nucleotide position in the wild-type heterozygous father, heterozygous mother, heterozygous sister, heterozygous brother, and homozygous patient. (**D**) The alignment of the conserved Leu1959 residues from different DYSF protein orthologs was compared using the CLUSTALW server. The Leu amino acids are shown in the box. (**D**) The image shows the functions and protein interactions of the distinct DYSF regions. The scheme summarizes some of the roles described for different DYSF C2 domains. (**E**) The image presents a comparison of the interactions between the normal DYSF and the Leu1959Pro variant and calpain-3, annexin A1, and affixin

DNA was isolated using the DNSol Midi kit (Roche: Product No. 50,072,012) from the peripheral blood samples of all the patient’s family members. The quality and quantity of the DNA samples were controlled using a NanoDrop spectrophotometer. WES (library construction, capture, and sequencing) was conducted on the proband (Fig. 2A, III-1) at Macrogen (Amsterdam, Netherlands). Exome capture was performed using an XT Library Prep kit. The exome library preparation was then sequenced on the Illumina HiSeq 6000 platform, generating 100-bp paired-end reads at an average depth of approximately 100×. For each sample, a depth of greater than 7 and a read quality above 20 were required for consideration. The initial quality control of the raw sequencing data was carried out using FastQC. The reads were then aligned to the GRCh37 human reference genome using the Burrows-Wheeler Aligner (BWA-MEM v0.7.17) [9]. The Genome Analysis Toolkit (GATK, v.4.1.4.1) was used to perform single-nucleotide polymorphism and intra-read insertion and deletion calling [10]. Genetic variants were annotated and filtered using ANNOVAR [11] based on genomic positions and minor allele frequencies below 0.005 in the 1000 Genomes Project, ExAC, gnomAD, and ESP6500. Additionally, variants outside of exonic sequences and synonymous alterations were disregarded. The remaining non-synonymous variants were subjected to bioinformatics analysis.

#### Bioinformatics analysis

Stringent criteria were applied to confirm the candidate variants by bioinformatics tools, such as CADD (phred > 15) (cadd.gs.washington.edu/), SIFT (score ≤ 0.05: deleterious and score > 0.05 tolerable) (https://sift.bii.a-star.edu.sg), PolyPhen-2 (score = 0–0.15: benign; score = 0.15–0.85: possibly damaging; and score = 0.85–1: probably damaging) (genetics.bwh.harvard.edu/pph2), MutationTaster (www.mutationtaster.org), PROVEAN (score ≤ − 2.5: deleterious and score > − 2.5: natural) (provean.jcvi.org), and according to the American College of Medical Genetics and Genomics 2015 (ACMG) guidelines [12]. Variations identified as harmful by 2 prediction tools were chosen. The analysis for conservational changes in protein positions involved a comparison of amino acid sequences from various species, conducted using the CLUSTALW web server (https://www.genome.jp/tools-bin/clustalw).

#### Validation of the identified variant

Following bioinformatics assessment, segregation analysis via Sanger sequencing was performed for all the proband’s family members. Primer pairs were designed using Gene Runner v6.0 software with the following sequences: forward:5′-TGAACCTGCCCTGCCTAA-3′.

and reverse: 5′- GATGTGAGCCGCAAAGGA-3′. A polymerase chain reaction (PCR) mix consisting of 1.5 mmol/L of MgCl2, 200 mmol/L of dNTP, 10 pmol/L of primers, 1 U Taq DNA polymerase (Amplicon, UK), and 100 ng of the genomic DNA template was prepared. The PCR thermal cycling conditions were 95 °C for 5 min (35 cycles of 95 °C for 30 s, 64 °C for 30 s, and 72 °C for 30 s) and final extension at 72 °C for 10 min. The PCR products were sequenced on an ABI Sequencer 3500XL (Applied Biosystems) and analyzed using FinchTV 1.4.0 (Fig. 2B).

### Modeling and docking studies

#### DYSF and binding partners associated with cell membrane repair in skeletal muscles

DYSF is an approximately 240-kDa protein comprising seven C2 domains along with additional ferlin-like domains, FER and DYSFv, and a C-terminal transmembrane domain [13]. It is abundantly expressed in skeletal muscle transverse tubules, suggesting its involvement in membrane repair [14]. The process of DYSF-mediated membrane repair is vital for maintaining the integrity of cell membranes, particularly in skeletal muscle cells [13]. In healthy muscle fibers, DYSF is localized both at the plasma membrane and within cytoplasmic vesicles. Upon damage to a muscle cell membrane caused by mechanical stress or injury, extracellular calcium influx occurs, followed by the recruitment of DYSF to the damaged site. DYSF then interacts with other proteins, forming a complex essential for initiating membrane repair. Notable proteins within this complex include mitsugumin-53, annexin A1, annexin A2, caveolin-3, calpain-3, syntaxin-4, AHNAK1, and affixin [15]. The DYSF-mediated membrane repair process involves several steps. Initially, DYSF moves to the injury site, binding to phospholipids on the damaged membrane, thereby accumulating at the site [16]. Subsequently, DYSF facilitates the recruitment and fusion of intracellular vesicles containing membrane components, derived from the endoplasmic reticulum and the trans-Golgi network with the impaired membrane. This fusion is aided by interactions between DYSF with other proteins, such as mitsugumin-53. Once fusion occurs, newly inserted membrane components help restore the structural integrity and functionality of the muscle cell membrane [15], essential for maintaining normal muscle function and preventing degeneration [17]. The C2 domains of DYSF play a crucial role in its membrane repair function in skeletal muscles [18]. These domains possess calcium-binding properties and are involved in various cellular processes, including membrane fusion and vesicle trafficking. Proteins involved in the repair process bind to the C2 domains of DYSF, facilitating the recruitment and fusion of vesicles containing membrane material to the damaged site in skeletal muscle cells. This interaction is pivotal for the repair process, ensuring the integrity of the injured membrane. DYSF is an approximately 240-kDa protein and contains 7 C2 domains with additional ferlin-like domains, FER and DYSFv, and a C-terminal transmembrane domain. Considering that the identified variant was located in the C-terminal domain of DYSF, we investigated the docking of the C-terminal, comprising C2G and TM domains, with partners involved in binding during the repair process. Specifically, DYSF C2G interacts with caveolin-3 and syntaxin-4 during resting conditions and with calpain-3, annexin A1, and affixin during the repair process [15].

#### Modeling and interaction studies

In the first stage, the 3D structures of DYSF (the normal DYSF and the Leu1959Pro variant), calpain-3, annexin A1, and affixin were predicted with AlphaFold2 (https://colab.research.google.com/github/sokrypton/ColabFold/blob/main/AlphaFold2.ipynb) [19]. Then, hydrogens were added, and structure optimization was charged using AutoDockTools software. Next, molecular docking analysis was conducted using the HADDOCK server (https://wenmr.science.uu.nl/haddock2.4/) [20]. Finally, molecular interactions were checked and viewed using PyMOL v.2.5.2 and LigPlus + v.2.2.4 [21].

## Results

### Clinical information

The proband, a 28-year-old Iranian adult, stood at a height of 184.3 cm and weighed 61 kg, measured using a wall-mounted stadiometer and a digital floor scale, respectively. His body mass index (BMI) was calculated at18.01 kg/m^2^. Waist circumference was measured 84 cm using a flexible tape positioned at the midpoint between the lowest palpable rib and the top of the iliac crest, while hip circumference was recorded at 94 cm, measured at the maximum circumference of the buttocks. The waist-to-hip ratio was calculated at 0.89. Upper arm circumference was 36 cm, measured at the mid-point between the acromion and the olecranon while standing with arms relaxed at the sides, and calf circumference of 30.1 cm taken at the point of the largest circumference while sitting with the knees flexed at 90°. All measurements were conducted on the right side of the body by a trained technician following standard anthropometric. The patient presented with progressive muscle weakness, particularly evident in the lower limbs, impacting daily activities. Physical examinations revealed symmetrical muscle wasting in the legs and pelvic girdle muscles, with progressive muscle weakness predominantly affecting the toes and the calf muscles. Elevated serum CK level were observed at 2901 U/L. Clinical data, electromyography results, and muscle biopsy all supported the diagnosis of LGMD. Due to dyspnea, the patient was referred to Rajaie Cardiovascular Medical and Research Center, Tehran, Iran. At the center, an echocardiographic examination revealed a decreased left ventricular ejection fraction (20%). Consequently, a cardiac magnetic resonance imaging test was recommended (Fig. 1B-C).

#### Muscle biopsy findings

Interpretation of the muscle biopsy from the patient’s right vastus lateralis proved challenging owing to marked atrophy and a prominent group atrophy pattern, suggesting neurogenic atrophy. Rare necrotic fibres were noted as associated with severe endomysial fibrosis (Fig. 1A). An immunohistochemical study revealed sarcolemmal labelling of muscle fibres except for the DYSF antibody (Fig. 1B).

#### Cardiac magnetic resonance imaging findings

The cardiac magnetic resonance imaging test showed a normal left ventricular size with a left ventricular end-diastolic volume indexed to the body surface area of 76 mL/m^2^, a moderately reduced left ventricular ejection fraction (45%), a normal right ventricular size with an end-diastolic volume indexed to the body surface area of 86 mL/m^2^, a mildly reduced right ventricular ejection fraction (43%), and no regional wall motion abnormalities in the ventricles (Supplementary Video 1). The short Tau inversion recovery sequences showed no inflammation or edema. The T1 mapping sequence demonstrated a T1 value in the mid-septal of 1040 milliseconds with a bullseye plot (Fig. 1C). The late gadolinium enhancement sequences illustrated no specific enhancement (Fig. 1D). Based on the imaging features of mild biventricular systolic dysfunction, a normal biventricular size, and no significant replacement fibrosis in the left ventricular myocardium, the proband’s phenotype was compatible with arrhythmogenic cardiomyopathy with biventricular involvement (Supplementary Table 1).

### Genetic findings

The proband (Fig. 2A, III-1) underwent a WES test. After filtration and prioritization, a single variant remained. It was a new pathogenic variant (NM_003494.4, c.5876T > C: p. Leu1959Pro) in exon 52 of *DYSF*. According to the ACMG guidelines, the c.5876T > C variant was classified as a pathogenic variant (criteria: PM2, PM4, PP1, PP3, and PP5). The c.5876T > C missense variant was not reported previously in 1000G, ExAC, Genome AD, HGMD, and ClinVar or publications. Via various prediction tools, such as CADD (phred = 29.4), SIFT (deleterious), PolyPhen-2 (probably damaging), MutationTaster (disease-causing), and PROVEAN (deleterious), the c.5876T > C missense variant was identified as the cause of the disease (Supplementary Table 2). The candidate variant was confirmed in the proband (Fig. 2B, III-1) in a homozygous state and was also present in his other available family members in a heterozygous state (Fig. 2B, II-3, II-4, III-2, & III-3). Furthermore, based on the outcomes from CLUSTALW, Leu1959 was identified within the conserved region of DYSF (Fig. 2C).

### Modeling and docking analysis

**Fig. 3:**
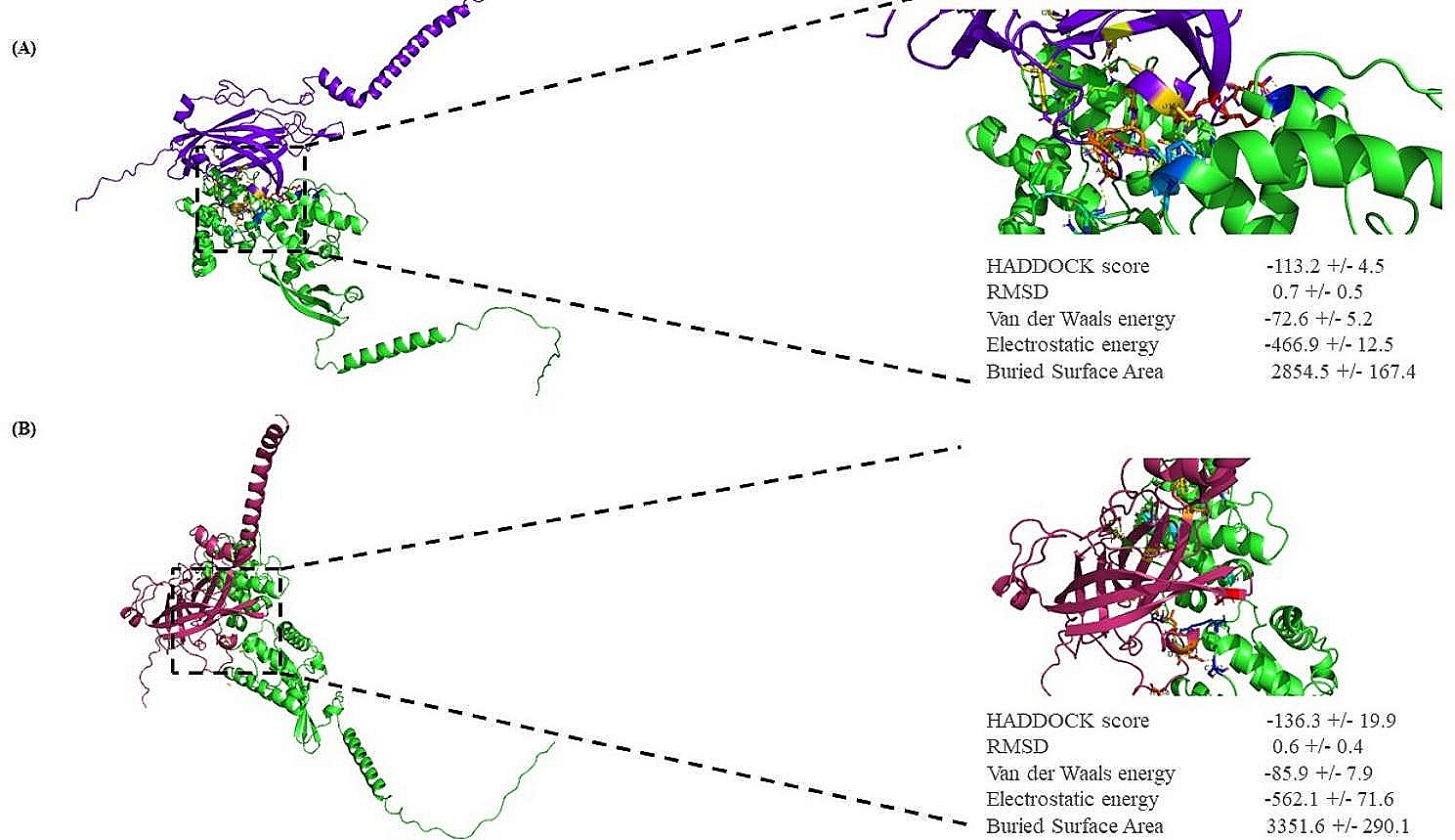
A molecular docking examination of the normal DYSF and the Leu1959Pro variant interacting with affixin was conducted using PyMOL v.2.5.2. (**A**) The protein-protein interaction between the normal DYSF and affixin is displayed (affixin: green and the normal DYSF: purple). (**B**) The image illustrates the protein-protein interaction between the Leu1959Pro variant and affixin protein (affixin: green and the Leu1959Pro variant: pink)

**Fig. 4:**
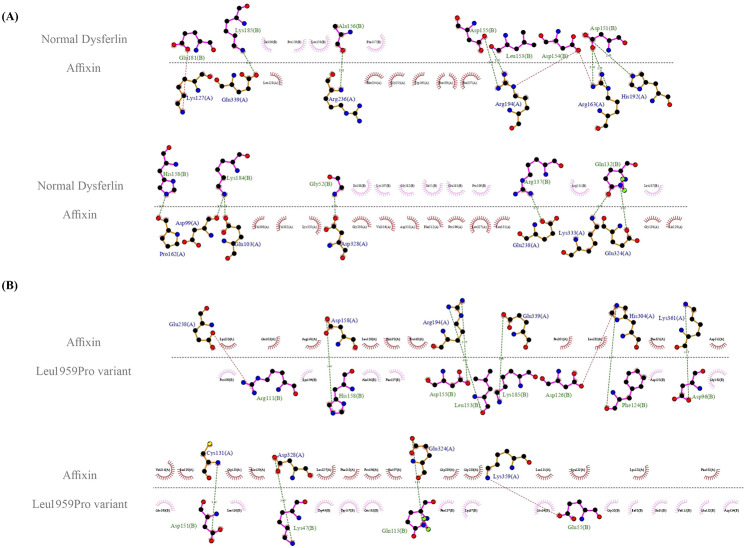
The image presents a schematic illustration of the best docking results of the interaction between the normal DYSF (**A**) and the Leu1959Pro variant (**B**) and affixin presented by LigPlus + v.2.2.4. Tee hydrogen bonding is in green, and the salt bridge is in red

AlphaFold2 was used to predict the structure of the C2G and TM domains (amino acids 1795–2080) of DYSF (Fig. 2D). The top-scoring models for both the normal DYSF and the Leu1959Pro variant were downloaded from AlphaFold2, with predicted local distance difference test scores of 87.6 and 86.9, respectively. The score ranges from 0 to 100, indicating how closely the model matches the true protein structure. A docking study was conducted using the modeled structures of the normal DYSF and the Leu1959Pro variant, along with affixin, annexin A1, and calpain-3 structures obtained from the AlphaFold Protein Structure Database (alphafold.ebi.ac.uk) [22, 23]. The first docking study was performed between DYSF (both the normal DYSF and the Leu1959Pro variant) and affixin. The HADDOCK score for the normal DYSF and the Leu1959Pro variant with affixin was − 113.2 ± 4.5 and − 136.3 ± 19.9, respectively, with root mean square deviation values of 0.7 ± 0.5 Å and 0.6 ± 0.4 Å. The normal DYSF formed 14 hydrogen bonds, while the Leu1959Pro variant produced 9 hydrogen bonds (Fig. 3A and B). The selected docked cluster of DYSF showed several close interactions with affixin involving residues such as Gly1846, Gln1926, Arg1931, Asp1945, Leu1947, Asp1948, Asp1949, Ala1950, His1952, Glu1975, Lys1978, and Lys1979. However, in the Leu1959Pro variant model, Lys1841, Glu1849, Asp1890, Arg1905, Gln1909, Phe1918, Asp1920, Asp1945, Leu1947, Asp1949, His1952, and Lys1979 were engaged in these interactions (Fig. 4A and B).

**Fig. 5:**
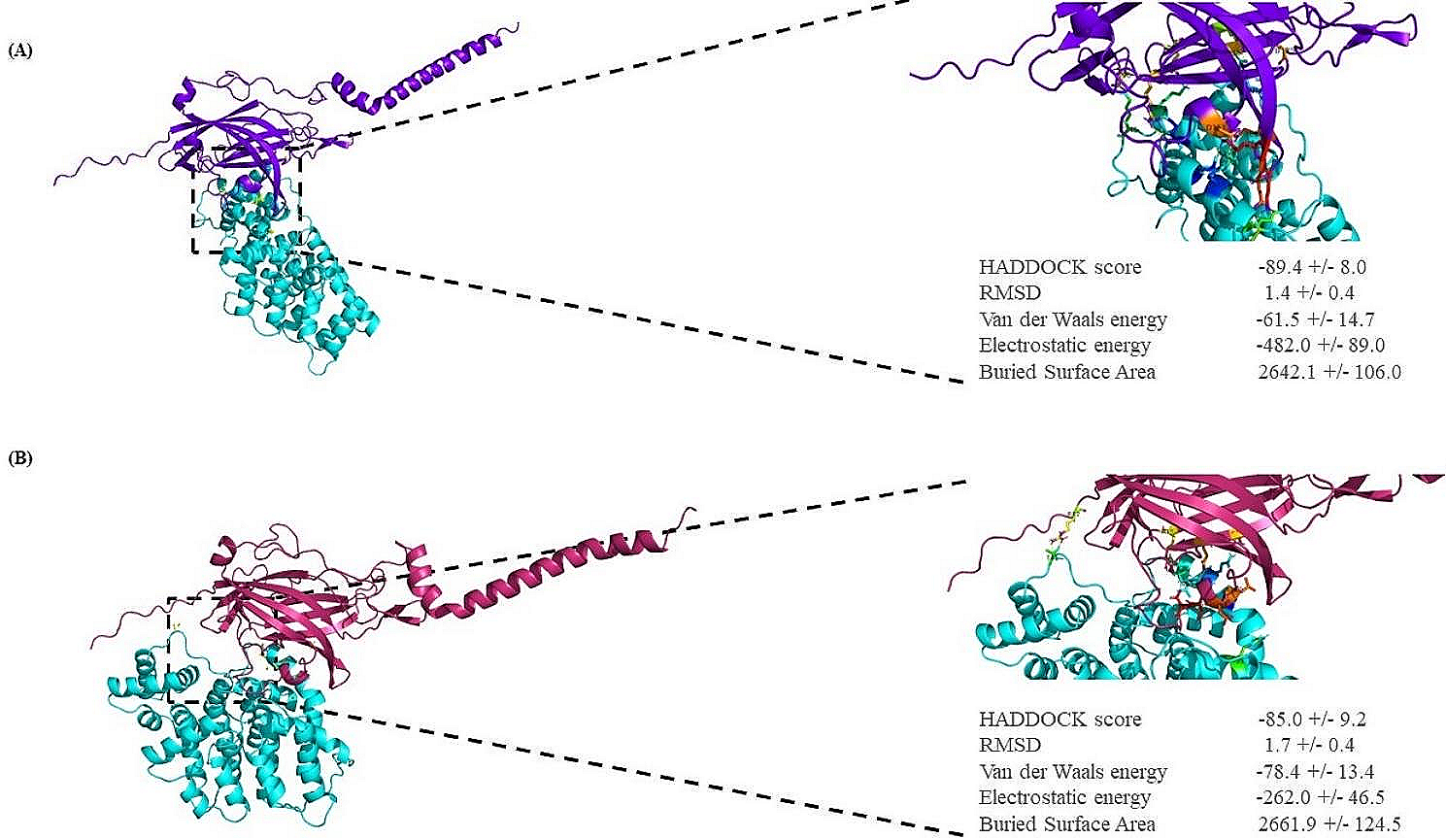
A molecular docking examination of the normal DYSF and the Leu1959Pro variant interacting with annexin-A1 was conducted using PyMOL v.2.5.2. (**A**) The protein-protein interaction between the normal DYSF and annexin-A1 is displayed (annexin-A1: blue and the normal DYSF: purple). (**B**) The image displays the protein-protein interaction between the Leu1959Pro variant and annexin-A1 (annexin-A1: blue and the Leu1959Pro variant: pink)

**Fig. 6:**
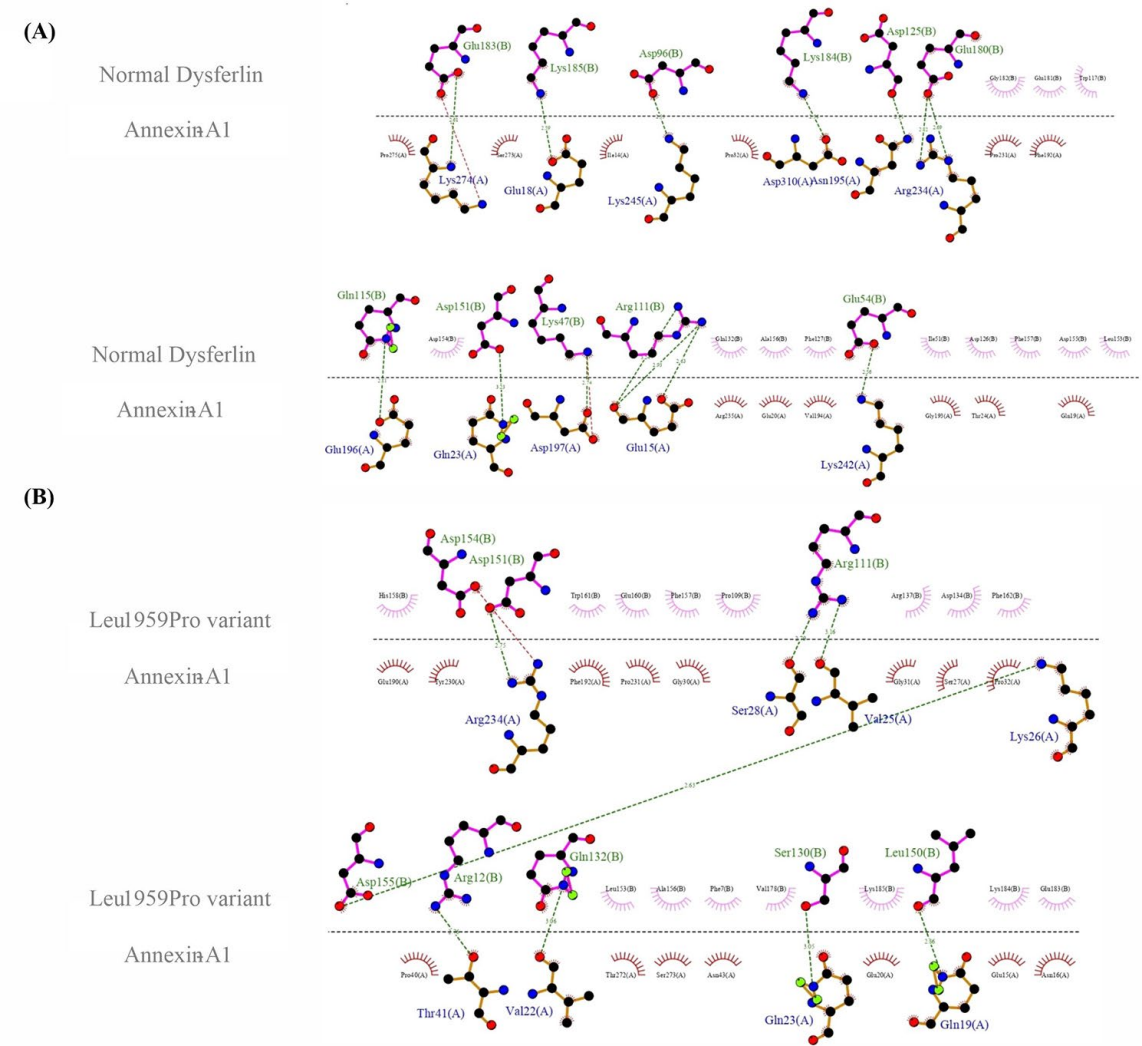
The image presents a schematic illustration of the best docking results of the interaction between the normal DYSF (**A**) and the Leu1959Pro variant (**B**) and annexin-A1 presented by LigPlus + v.2.2.4. The hydrogen bonding is in green, and the salt bridge is in red

**Fig. 7:**
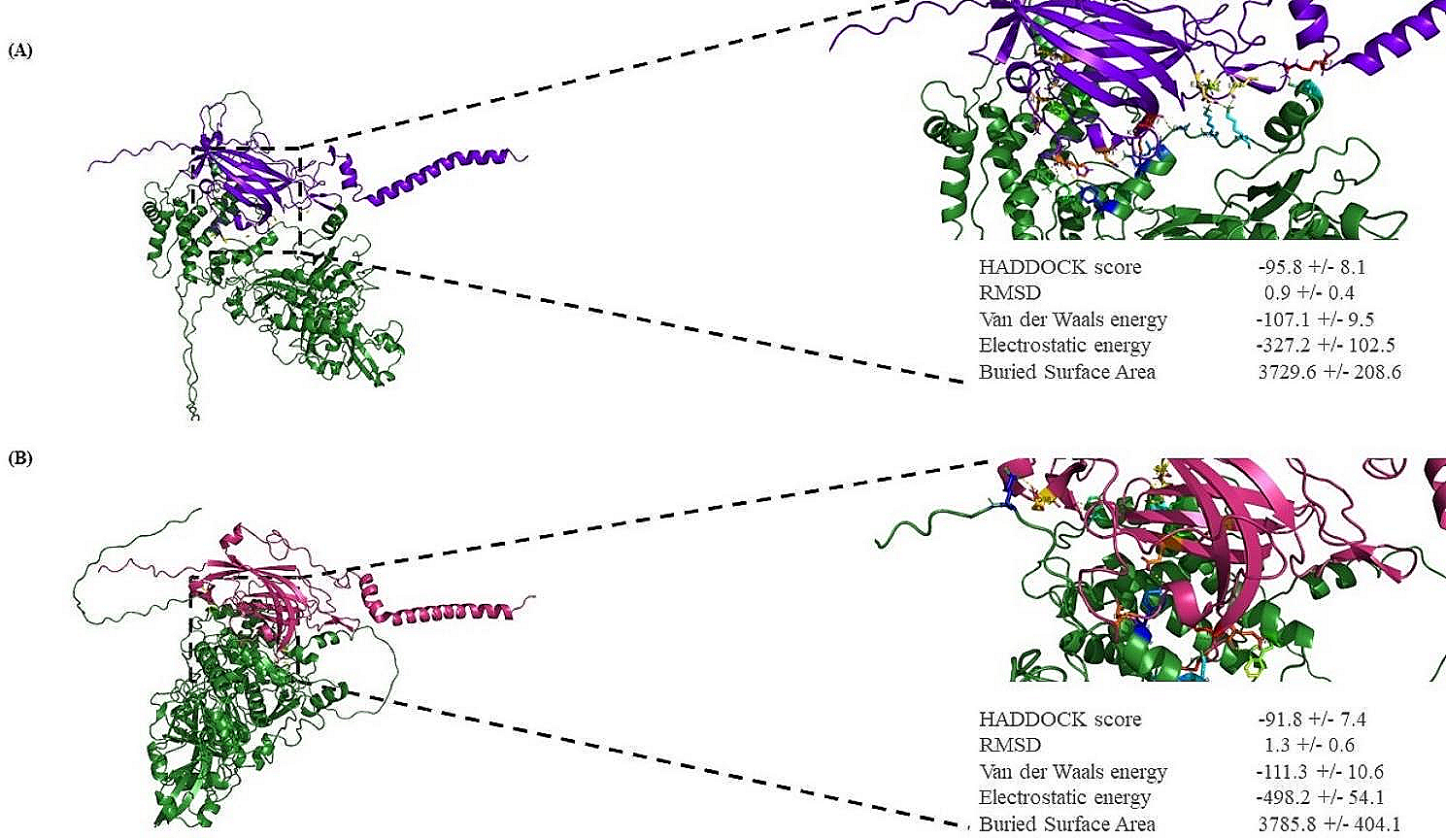
A molecular docking examination of the normal DYSF and the Leu1959Pro variant interacting with calpain-3 was conducted using PyMOL v.2.5.2. (**A**) The image shows the protein-protein interaction between the normal DYSF and calpain-3 (calpain-3: deep green and the normal DYSF: purple). (**B**) The image illustrates the protein-protein interaction between the Leu1959Pro variant and calpain-3 (calpain-3: deep green and the Leu1959Pro variant: pink)

**Fig. 8:**
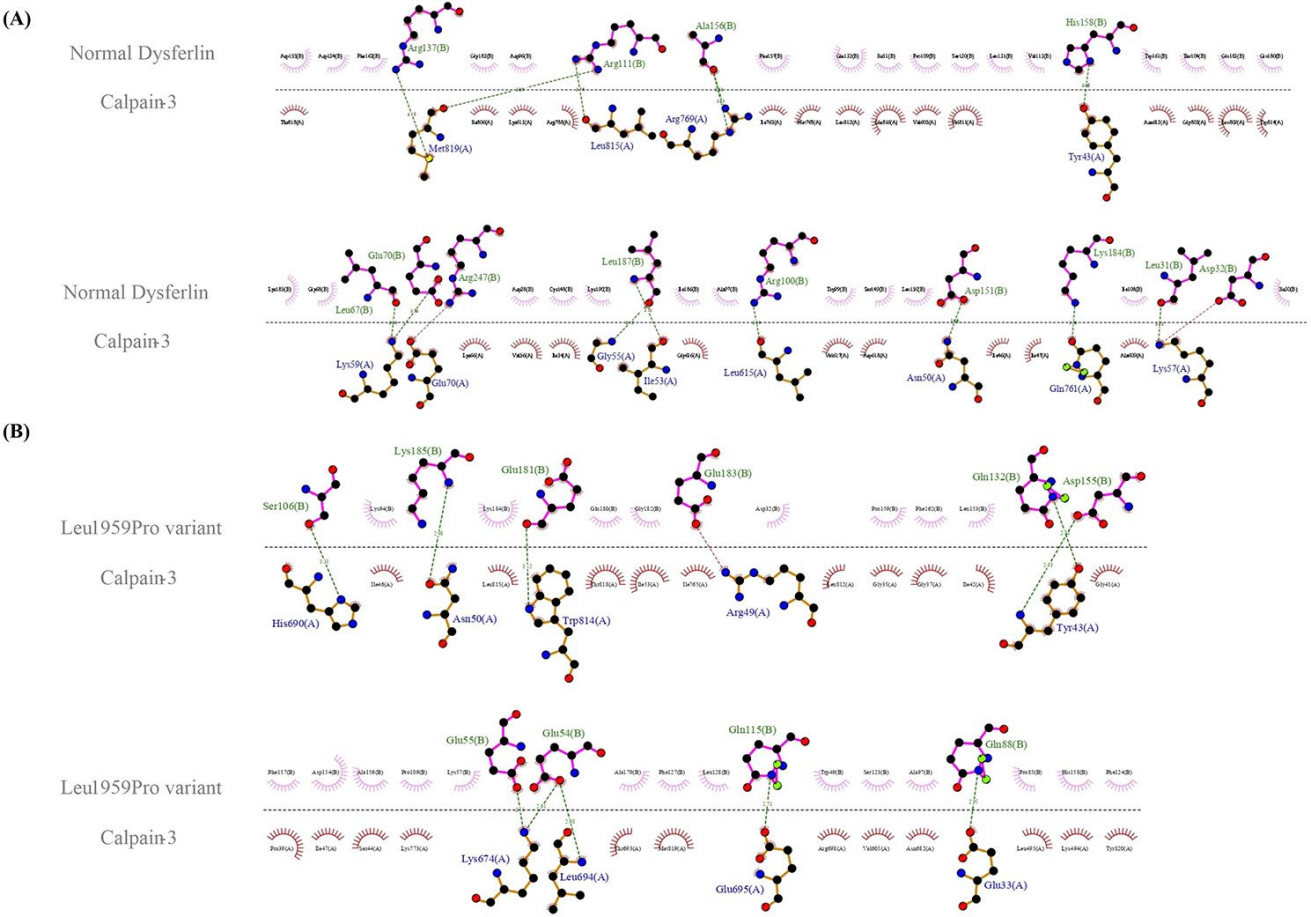
The image presents a schematic illustration of the best docking results of the interaction between the normal DYSF (**A**) and the Leu1959Pro variant (**B**) and calpain-3 presented by LigPlus + v.2.2.4. The hydrogen bonding is in green, and the salt bridge is in red

The second docking study was carried out between DYSF (both the normal DYSF and the Leu1959Pro variant) and annexin A1. The HADDOCK score for the Leu1959Pro variant with annexin A1 was − 89.4 ± 8.0 and − 85.0 ± 9.2, respectively, with root mean square deviation values of 1.4 ± 0.4 Å and 1.7 ± 0.4 Å (Fig. 5A and B). The normal DYSF formed 14 hydrogen bonds with annexin A1, while the Leu1959Pro variant produced 8 hydrogen bonds. The residues involved in the normal DYSF form were Glu1849, Asp1890, Arg1905, Gln1909, Asp1919, Lys1941, Asp1945, Glu1974, Glu1977, Lys1978, and Lys1979, while those in the Leu1959Pro variant were Arg1806, Arg1905, Ser1924, Gln1926, Leu1944, Asp1945, Asp1948, and Asp1949 (Fig. 6A and B). The HADDOCK scores for the bonding between the normal DYSF and the Leu1959Pro variant and calpain-3 in the final docking study were − 95.8 ± 8.1 and − 91.8 ± 7.4, respectively, with root mean square deviation values of 0.9 ± 0.4 Å and 1.3 ± 0.6 Å (Fig. 7A and B). The normal DYSF generated 13 hydrogen bonds with calpain-3, whereas the Leu1959Pro variant formed 10 hydrogen bonds. The Leu1825, Asp1826, Leu1861, Glu1864, Arg1894, Arg1905, Arg1931, Asp1945, Ala1948, His1952, Leu1981, Lys1978, and Arg2041 were involved in the bonding between the normal DYSF and calpain-3, while Glu1848, Glu1849, Gln1882, Ser1900, Gln1909, Gln1926, Asp1949, Glu1975, Glu1977, and Lys1979 were engaged in the bonding between the Leu1959Pro variant and calpain-3 (Fig. 8A and B). All 3 docking studies revealed that the Leu1959Pro variant had a lower binding affinity with affixin, annexin A1, and calpain-3 than the normal DYSF (Fig. 2E).

## Discussion

LGMD is a genetic disorder characterized by progressive muscle weakness and dystrophy, primarily affecting the proximal muscles. Dysferlinopathy exhibits a high prevalence among LGMD patient cohorts in regions or communities with high consanguinity rates, such as the Maghreb, Israel, Saudi Arabia, Egypt, Iran, and India [6]. In a cohort of 300 Indian patients diagnosed with LGMD, dysferlinopathy was determined in 33% of cases based on the detection of deficient DYSF protein levels [24]. In the present study, we identified a homozygous *de novo* likely pathogenic variant in *DYSF* (c.5876T > C: p. Leu1959Pro) causing LGMD. This variant was completely segregated within the proband’s family. Variants in *DYSF* can lead to dysferlinopathy, a subgroup of LGMD [25]. Several studies have also investigated *DYSF* variants and their association with muscular dystrophy.

A study on an Israeli family observed the onset of vision problems and nystagmus in early childhood, along with LGMD arising from this variant. However, our patient did not exhibit these issues [26]. Lorenzoni et al. [27] reported 36 cases with this variant and muscle dystrophy but with no cardiac abnormalities. In contrast, in our study, an echocardiogram showed a decreased left ventricular ejection fraction (20%), indicating cardiac involvement. Bansal et al. [28] demonstrated defective membrane repair in DYSF-deficient muscular dystrophy. WES investigations have also confirmed that *DYSF* variants account for between 16% and 23% of molecular diagnoses in patients with LGMD [29]. In a study conducted in Poland to sample the European population, 50 out of 72 cases with LGMD (68.5%) were found to have pathogenic variants [8]. In cases suspected of having dysferlinopathy either clinically or pathologically, NGS has aided in identifying *DYSF* variants in 36% [30]. Accurate diagnosis of LGMD is crucial for appropriate management and long-term prognosis [31]. DYSF, accompanied by other interacting proteins, plays a crucial role in recruiting vesicles and facilitating their fusion with damaged cell membranes to restore integrity and maintain cellular function [14]. DYSF-mediated membrane repair can become impaired or dysfunctional in genetic disorders known as dysferlinopathy [13]. When a muscle cell membrane is injured, DYSF is recruited to the site of damage, where it interacts with other proteins to form a complex that initiates membrane repair [32]. This repair complex includes proteins such as mitsugumin-53, annexin A1, annexin A2, caveolin-3, calpain-3, syntaxin-4, AHNAK1, and affixin [15]. To investigate the repair process, prior studies assessed docking between the C-terminal domain of DYSF (including the C2G and TM domains) and calpain-3, annexin A1, and affixin. The docking analysis revealed that this specific variant was associated with reduced binding between DYSF and calpain-3, annexin A1, and affixin, exerting a dominant-negative impact on the role of DYSF in the membrane repair process. Identifying disease-causing *DYSF* mutations has enabled several key clinical applications. Definitive genetic diagnosis of dysferlinopathy now allows for specialized monitoring approaches focused on expected symptoms [33, 34]. Additionally, elucidating the underlying molecular mechanism introduces novel targets for possible gene and cell-based therapies [33]. Other cutting-edge strategies aim to augment DYSF expression through exon-skipping or CRISPR editing [35]. These reproductive options provide families with a degree of control over the inheritance of the condition. Still, although significant progress is being achieved in the management of dysferlinopathy, there is still more work to be done in order to transition potential treatments from preclinical research to human trials [36]. Indeed, the discovery of DYSF variants and the resulting capacity to offer genetic diagnosis remains the essential first step in enabling all future clinical advancements for this form of muscular dystrophy [37].

## Conclusion

The current study identified a *de novo* likely pathogenic variant in *DYSF* causing LGMD. The detection of this variant underscores the utility of NGS in diagnosing and delineating the genetic underpinnings of LGMD. Further research is warranted to elucidate the functional repercussions of this variant and its effects on the function of DYSF.

### Electronic supplementary material

Below is the link to the electronic supplementary material.

**Supplementary Material 1: Supplementary Table 1.** Clinical information of the studied patient

**Supplementary Material 2: Supplementary Table 2.** Identified variant in this study

**Supplementary Material 3: Supplementary Video.** The four-chamber cine image showing mild bi-ventricular dysfunction and normal-size ventricles

## Data Availability

The data sets generated and/or analyzed during the current study are available in the ClinVar repository [https://www.ncbi.nlm.nih.gov/clinvar/variation/2506389/]. The accession number of the variant in ClinVar is as follows: NM_001130987.2 (*DYSF*): c.5993T > C (p. Leu1998Pro): VCV002506389.1.
