# Supplementary material for: A novel homozygous variant (c.5876T > C: p. Leu1959Pro) in DYSF segregates with limb-girdle muscular dystrophy: a case report
Source: BMC Musculoskelet Disord. 2024 Mar 27;25:241. doi: 10.1186/s12891-024-07354-9 (PMC10967161; doi:10.1186/s12891-024-07354-9)
Supplement: Supplementary file 1 — Supplementary Material 1: Supplementary Table 1. Clinical information of the studied patient [file 12891_2024_7354_MOESM1_ESM.docx]

| Supplementary Table 1. Clinical information of the studied patient. | | | | |
| --- | --- | --- | --- | --- |
| Patient | **Age** | **Clinical findings** | | |
|  |  | **Symptoms** | **Muscle biopsy** | **Cardiac magnetic resonance** |
| Male | 28-year-old | - Weakness   The weakness began bilaterally in the toes and progressed to his calf muscles and the distal regions of the lower limbs.   - Some degree of dyspnea | - Rare necrotic fibers were noted associated with severe endomysial fibrosis. - Immunohistochemical study revealed sarcolemmal labelling of muscle fibers except with dysferlin antibody. | The phenotype was compatible with arrhythmogenic cardiomyopathy (ACM) with biventricular involvement |
|  | | | | |
