# Supplementary material for: A novel homozygous variant (c.5876T > C: p. Leu1959Pro) in DYSF segregates with limb-girdle muscular dystrophy: a case report
Source: BMC Musculoskelet Disord. 2024 Mar 27;25:241. doi: 10.1186/s12891-024-07354-9 (PMC10967161; doi:10.1186/s12891-024-07354-9)
Supplement: Supplementary file 2 — Supplementary Material 2: Supplementary Table 2. Identified variant in this study [file 12891_2024_7354_MOESM2_ESM.docx]

| Supplementary Table 2. Identified variant in this study. | | | | | | | | |
| --- | --- | --- | --- | --- | --- | --- | --- | --- |
| Gene/transcript | **Variant**  **location** | **Variant** | **CADD** | **SIFT** | **PolyPhen-2** | **MutationTaster** | **PROVEAN** | **ACMG** |
| *DYSF*  NM_003494.4 | Exon 52 | c.5876T>C  p. Leu1959Pro | 29.4 | Deleterious | Probably damaging | Disease causing | Deleterious | Pathogenic |
|  | | | | | | | | |
